# In Vitro and Ex Vivo Models to Study Molecular Trafficking Across the Human Intestinal Barrier

**DOI:** 10.3390/ijms262110535

**Published:** 2025-10-29

**Authors:** Andrea Galvan, Elsa Guidorizzi, Flavia Carton, Manuela Malatesta, Laura Calderan

**Affiliations:** 1 Department of Neurosciences, Biomedicine and Movement Sciences, University of Verona, 37134 Verona, Italy; andrea.galvan@univr.it (A.G.); elsa.guidorizzi@studenti.univr.it (E.G.); laura.calderan@univr.it (L.C.); 2 Center for Medical Sciences (CISMed), University of Trento, 38122 Trento, Italy; flavia.carton@unitn.it; 3 Department of Cellular, Computational and Integrative Biology (CIBIO), University of Trento, 38123 Trento, Italy

**Keywords:** intestinal absorption, intestine model, artificial membrane, cell culture, 3D bioprinting, organ-on-a-chip, intestine explant

## Abstract

The intestine is a complex organ whose main functions are food digestion and nutrient absorption. It is therefore of great interest for pharmaceutical research as a preferred route for drug delivery. In vitro intestinal models are valuable tools for the preclinical evaluation of absorption, distribution, metabolism, and excretion of new therapeutic formulations; consequently, several attempts have been made to recreate the human intestine barrier in vitro. The models so far set up were aimed at mimicking specific intestinal features related to the molecules or processes under investigation. Artificial membranes are suitable to study passive absorption; systems based on 2D/3D cell cultures reproduce the transcellular pathway; organs-on-a-chip mimic the in vivo cellular and mechanical complexity, allowing the identification of the multiple factors involved in molecular interactions with the intestinal barrier; and intestine explants replicate in full the native organ under controlled conditions, thus providing the most comprehensive in vitro model. All these models have advantages and disadvantages but all have given important contribution to advance the knowledge on the interaction of drugs, toxins, and xenobiotic with the intestinal barrier.

## 1. Introduction

The intestine is a complex organ whose main functions are food digestion and nutrient absorption, although it is also involved in exocrine and endocrine secretion and in immune defense [1,2]. The intestine is therefore a main target in pharmaceutical research since the oral administration is the preferred route for drug delivery thanks to its ease of use and non-invasiveness [3]. In fact, the intestine plays a key role in absorption and first-pass metabolism of drugs and represents a primary site for potential off-target toxicity. Understanding the mechanisms that regulate the molecular absorption and the effects on the intestinal barrier is therefore crucial.

The strict rules concerning the work with laboratory animals imposed by the 3R (Replacement, Reduction and Refinement) principle [4] together with the need of experimental results directly applicable to humans bypassing interspecific differences have oriented researchers towards in vitro models of human intestine. In vitro intestinal models are in fact valuable tools for the preclinical evaluation of ADME (absorption, distribution, metabolism, and excretion) parameters, and for optimizing the bioavailability and safety of new therapeutic formulations. In addition, the effect of specific molecules on the intestinal barrier is challenging to investigate in the whole organism, while it is much easier to manage under simplified in vitro conditions [1].

However, reproducing in vitro the structural and functional features of the human intestinal barrier is a hard challenge.

### 1.1. Structural and Functional Features of the Human Intestinal Barrier

**Figure 1:**
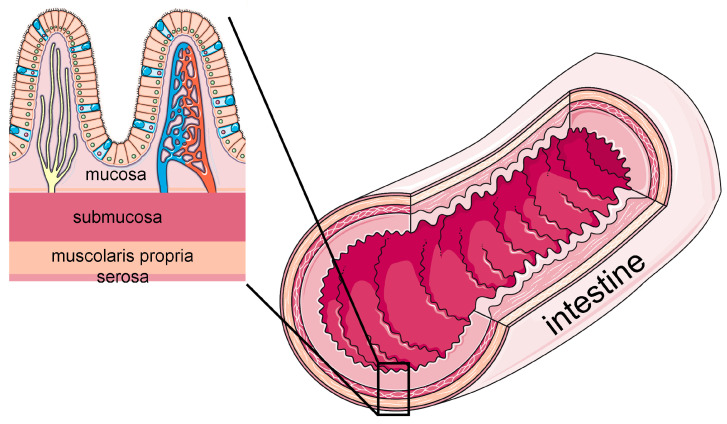
Schematic representation of the four layers composing the intestinal wall. Modified from images provided by Servier Medical Art (https://smart.servier.com/), licensed under CC BY 4.0 (https://creativecommons.org/licenses/by/4.0/) (accessed on 28 September 2025).

The intestine is an extensive tubular organ divided into two main segments, i.e., the small and large intestine. The small intestine is in continuity with the stomach proximally and with the large intestine distally and is divided into three regions (i.e., duodenum, jejunum, and ileum), each of them characterized by a wall with multiple folding (plicae, villi, and cell microvilli) ensuring an enormous absorption surface. The large intestine is divided into four regions (cecum, colon, rectum, and anal canal) and is characterized by a limited wall folding. The small and large intestine play distinct functional roles in absorption, secretion, and immune activity and are even characterized by different microbial populations [5]. Both segments consist of four concentric tissue layers: the innermost, facing the organ lumen, is called mucosa and covers the sub-mucosa, which is made of connective tissue abundant in blood and lymphatics vessels; below the sub-mucosa lies the muscularis propria, composed of two perpendicularly oriented layers of smooth muscle cells that are responsible for the peristaltic movements; the fourth, outermost layer is the serosa, a thin epithelial membrane that envelops the whole organ. (Figure 1). The intestinal mucosa is primarily responsible for the intestine barrier functions, playing a crucial role in the selective absorption of nutrients and in the defense against pathogens, toxins, and xenobiotics [6]. Structurally, it consists of three main components: the luminal tissue is the intestinal epithelium, which covers the connective lamina propria where a network of blood and lymphatic vessels and various immune cells are located; the outer layer is the muscularis mucosae made of concentrically oriented smooth muscle cells [7].

The intestinal epithelium is the most functionally active tissue, being directly involved in nutrient uptake and in providing chemical and immunological protection against the luminal environment [8]: it consists of a monolayer of polarized cells lining the whole intestinal lumen and includes various cell types with specific functions. Enterocytes, the predominant absorptive cells, feature an apical brush border composed of microvilli, which significantly increase the absorptive surface area and host numerous digestive enzymes and membrane transporters, such as peptide transporter 1 (PEPT1) and P-glycoprotein (P-gp), which are essential for nutrient and drug absorption. Goblet cells are specialized in secreting mucins and antimicrobial peptides, contributing to the formation of the protective mucus layer covering the enterocytes. At the base of the villi, the glandular structures called intestinal crypts or crypts of Lieberkühn contain several epithelial cells among which are the Paneth cells (that release antimicrobial peptides playing a key role in host defense and in maintaining the stem cell niche) and different enteroendocrine cells (that secrete peptide hormones regulating intestinal motility and the absorptive processes) [9]. At the base of the crypts, a population of intestinal stem cells are responsible for the continuous renewal of the epithelium; these cells actively proliferate and progressively migrate toward the villus apex, undergoing differentiation into the various epithelial cell types [10].

The mucosal layer is the main component of the intestinal barrier, whose primary function is to act as a selectively permeable interface that enables the efficient absorption of nutrients, electrolytes, and water, while simultaneously preventing the translocation of pathogens, toxins, and xenobiotics from the intestinal lumen into the underlying tissues and the systemic circulation. The molecular trafficking is tightly regulated by a complex system of intercellular junctions that maintain epithelial cohesion and control selective permeability. Tight junctions, located at the apical region of the cell lateral membrane, are composed of several families of transmembrane proteins (namely occludin, claudins, junctional adhesion molecules, and tricellulin) that interact to form a semi-permeable seal that dynamically regulates the passage of ions and small molecules among cells [8]. Beneath the tight junctions, adherens junctions reinforce the intercellular adhesion through cadherin-mediated interactions, stabilized by intracellular proteins that link the junctional complex to the actin cytoskeleton, thus contributing also to signal transduction and cytoskeletal remodeling. Desmosomes, composed of dense protein plaques connected to intermediate filaments, provide further mechanical integrity, allowing the epithelial layer to withstand shear stress and mechanical deformation.

Multiple transport mechanisms are involved in molecular trafficking across the intestinal barrier. Molecules may cross the epithelium via the transcellular route, moving directly through epithelial cells by passive diffusion, facilitated diffusion, or active transport via specific membrane transporters. Notably, passive diffusion through the lipid bilayer plays a significant role for small lipophilic compounds [11]. Alternatively, molecular transport across the intestinal barrier may occur via the paracellular route, which involves the passive movement of solutes between adjacent epithelial cells. Larger molecules, such as peptides, antigens, or immunoglobulins, are transported through endocytosis or transcytosis, involving vesicle-mediated mechanisms [8].

Once molecules have crossed the epithelial barrier, they must overcome additional barriers located in the mucosal lamina propria before reaching the systemic circulation, i.e., the gut vascular barrier, composed of the endothelial cells of blood vessels, pericytes, and enteric glial cells [6,12], and the lymphatic vessel barrier, whose endothelial cells allow for highly selective permeability to lipids, cholesterol, and lipoproteins [13].

### 1.2. Scope of the Review

Several attempts have been made to recreate the human intestine barrier in vitro, the complexity degree of the models varying with respect to the purpose of the study. In principle, all the histological layers composing the human intestine or, at least, all the cellular and extracellular components of the intestinal mucosa should be present in an ideal in vitro model of intestinal barrier. Moreover, the model should ensure optimal supply of oxygen and nutrients as well as mechanical stimuli to mimic in vivo environment and reliably reproduce the physiological functions [1,14].

The present article aims to provide a critical overview of the main in vitro models of the human intestine barrier currently available for studying the molecular trafficking and effects. The literature survey was made through the Scopus and PubMed databases, favoring papers published no more than ten years ago.

The presentation has been organized according to the increasing biological and technological complexity of the barrier models, and their reliability in recapitulating the structural and functional features of the human intestine has been discussed.

## 2. Artificial Membranes

**Figure 2:**
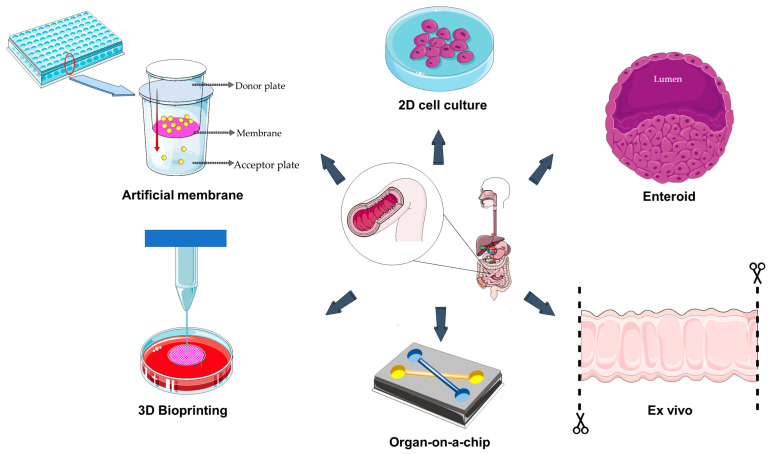
In vitro models of intestinal barrier. Modified from images provided by Servier Medical Art (https://smart.servier.com/), licensed under CC BY 4.0 (https://creativecommons.org/licenses/by/4.0/) (accessed on 28 September 2025).

In permeation studies across biological barriers, artificial membranes represent a helpful starting screening tool, helping researchers to identify the most promising candidates from a broad panel of molecules for further evaluation in subsequent research stages. For instance, the parallel artificial membrane permeability assay (PAMPA) has a wide spectrum of application and has been exploited for the high-throughput screening of molecules passively absorbed through the gastrointestinal tract [15]. This well-established system is based on the presence of an acceptor and a donor plate assembled in a sandwich-like structure where molecules must pass a hydrophobic filter (Figure 2) [16].

The compounds to be tested are generally solubilized in an aqueous donor medium and, after a defined incubation time, the proportion of the compound that has passed through the artificial lipid membrane is measured by ultraviolet-visible absorption or measurement with liquid chromatography/mass spectrometry (LC/MS) to determine the effective passive permeability coefficient [17,18]. For instance, Park and collaborators used a PAMPA system to assess the gastrointestinal tract permeability for different compounds used as preservatives, food contact applications, plastic raw materials, and biocides in consumer products potentially absorbed through the oral route such as parabens, bisphenols, isothiazolinones and phthalates [15]. From this study, the log P values (where P is the partition coefficient, describing how a compound is distributed between a polar aqueous phase and a non-polar organic phase) resulted to be the primary key factor delineating the permeability through the artificial membrane, with the permeability of alkyl ester parabens, bisphenols, and isothiazolinones resulting to be directly dependent on the log P values, while that of phthalates showed a reversed dependency. A more recent example of PAMPA application is the study of the passive gastrointestinal absorption of experimental antibacterial drugs, in particular dual DNA gyrase and topoisomerase IV inhibitors belonging to the *N*-phenylpyrrolamide, benzothiazoles, and the 4,5,6,7-tetrahydrobenzothiazole classes, quantifying these compounds with high-performance liquid chromatography-MS/MS [19]. This single-point analysis can be replaced by a real time analysis (RT-PAMPA), which offers multiple data points along the entire incubation period. An example is provided by He and coworkers, who developed a RT-PAMPA model administering a fluorescent artificial receptor into the acceptor well of PAMPA [18].

However, these artificial membranes provide data exclusively regarding passive transcellular mechanisms, while intestinal absorption relies also on other mechanisms such as the paracellular and active transports [17]. For this reason, in some studies the PAMPA system was associated with in vivo-like models. For instance, Marelli and coworkers tested the oral absorption of different enantiomeric pairs’ cyclic hexapeptides (three polar *N*-methylated cyclic hexaalanines, the lipophilic tri-*N*-methylated Veber–Hirschmann peptides, and the lipophilic peptide) using both PAMPA and Caco-2 cells (a cell line derived from a human colorectal adenocarcinoma) [20]. Exploiting the two systems, it was noticed that, even if different pairs of enantiomers had the same lipophilicities and PAMPA permeabilities, the Caco-2 permeability assay pointed out some differences. The results supported the idea that intestinal permeability of cyclic peptides, in particular the polar ones, may be influenced by a chiral carrier-mediated transport system, suggesting that the rational design of peptides with specific conformation and configuration could enhance their oral absorption. An interesting study by Berben and colleagues tested the predictivity of an artificial membrane insert (AMI)-system, consisting of a donor and an acceptor compartment separated by a regenerated cellulose membrane. The authors compared the permeabilities of fourteen different poorly water-soluble compounds covering a wide range of physicochemical parameters (e.g., ibuprofen, itraconazole, carvedilol, and naproxen) in both the AMI-system and in Caco-2 cells [21]. The study demonstrated a good correlation of the permeability coefficients between the two systems, pointing out the suitability of artificial membranes for an initial fast, simple, cost- and labor-effective assay of passive intestinal permeability of lipophilic compounds. Evolving the PAMPA concept, Kataoka and collaborators developed a double artificial membrane permeation assay (DAMPA) by which they compared the human intestinal permeabilities of different compounds (among which were metoprolol, ketoprofen, atenolol and pindolol) [16]. The author placed two artificial membranes, one in the apical and one in the basal side, with a compartment interposed, thus mimicking the double permeation process that normally occurs across the apical and basal membrane of enterocytes. This model proved to be predictive for passive absorption, and more time and cost effective in comparison to cell-based models.

Nevertheless, artificial membranes have limitations that cannot be overcome, as they are lacking the cellular components of the intestinal barrier as well as of the bacteria or molecular compounds that are physiologically present on the intestinal luminal surface. Danić and collaborators demonstrated that the presence of probiotic bacteria and bile acids in a PAMPA system affects the bioavailability of gliclazide [22]. More recently, the same group demonstrated that the permeability of PAMPA system for azathioprine was affected by probiotics and deoxycholate: drug permeability was significantly higher in the presence of the probiotic bacteria, potentially due to their metabolic activity, while deoxycholate caused a decrease [23]. In addition to the above-described limitations, there are other factors that may influence the outcome of PAMPA-based tests, such as incubation temperature, pH conditions and lipid membrane compositions, making the data obtained with this system not very predictable [17,24]. Despite these limitations, researchers are developing new membrane-based culture coated with components of the extracellular matrix in order to provide mechanical and biochemical cues able to support cell adhesion, growth, and differentiation into a mature epithelium [25]. Artificial membranes can also be improved by using a pre-made tissue constructs (EpiIntestinal^TM^ model) that include multiple cell types (epithelial, fibroblasts, and endothelial) cultivated on permeable membrane inserts in a multi-well format [26].

Table 1 summarizes the artificial membrane-based models of intestinal barrier described in this chapter.

## 3. Two-Dimensional and Three-Dimensional Cell Cultures

Both two-dimensional (2D) and three-dimensional (3D) cell culture models are currently used to study the human intestinal barrier; however, they differ in their capability to mimic the in vivo environment. Two-dimensional models consist of intestinal cells, such as Caco-2 cells, cultured as a monolayer on a rigid surface; under this condition, cells form intercellular junctions, thus generating a selectively permeable barrier (Figure 2). A classic model to study the passage of molecules across the intestinal barrier is made up of cells cultured on the porous membrane of transwell inserts [27]. This model allows the evaluation of transport mechanisms and the measurements of bidirectional permeability, i.e., the movement of a compound from the apical (upper) side to the basolateral (lower) side and vice versa [28]. In a recent study, Zhang et al. used transwell membranes to assess the oral uptake of epigallocatechin gallate through nanocarriers, investigating the uptake mechanisms and the bidirectional transport in Caco-2 cell monolayer [29].

Another system used for permeability studies across intestinal cells is represented by Ussing chambers. This system is composed of a cell monolayer seeded on a permeable support that divides the chamber into two distinct, isolated halves [30]. Park et al. used a Ussing chamber to investigate the protective effects of hesperidin on Caco-2 cell monolayers exposed to X-rays, demonstrating that this bioactive flavonoid was able to preserve transepithelial flux and permeability [31]. Bergmann et al. used a monolayer of T84 colon carcinoma cells grown in Ussing-type chambers to study how polyphenols are absorbed and affect the integrity of the gut barrier. They found that the ability of polyphenols to cross the barrier depended on their polarity. Additionally, some polyphenols, like ferulic and isoferulic acids, improved the barrier integrity by increasing the transepithelial electrical resistance (TEER) value [32].

Despite their suitability for some basic studies, cellular monolayers have limitations in mimicking the complexity of the intestinal barrier, such as the lack of interactions between different cell types, the presence of a mucus layer, and the expression of key metabolic enzymes [21,28,33]. To overcome these limitations, transwell models have been used in a co-culture setup where different cell types are grown in separate compartments to better resemble the in vivo cellular heterogenicity. For example, Strugari et al. developed a co-culture model of Caco-2 and HT29-MTX mucin-secreting cells (a human methotrexate-resistant colon cancer cell line) in transwell inserts to investigate the transport of silicon quantum dots and iron oxide nanoparticles. The results proved that neither the silicon quantum dots nor the iron nanoparticles were able to cross the Caco-2/HT29-MTX monolayer, probably due to their tendency to aggregate. Moreover, morphological alterations of some Caco-2/HT29-MTX co-cultures were observed, indicating the need for functionalization of nanoparticles and quantitative tests to assess their toxicity [34].

In drug efflux and passive permeability studies, Caco-2 monolayers in Ussing chambers have been complemented by human enteroids [33]. Enteroids are 3D “mini-intestines” created from intestinal crypt multipotent stem cells; they form a hollow, spherical structure with an inner lumen surrounded by an epithelial cell layer (Figure 2). Their capability to contain different cell types such as absorptive cells (enterocytes), mucus-producing cells (goblet cells), hormone-producing cells (enteroendocrine cells), and Paneth cells led researchers to create monolayers derived from enteroids. This involves breaking down the 3D enteroids into individual cells and then reseeding them on a flat, coated surface [33,35,36]. The generation of these monolayers represents the solution to the problem of the organoids’ enclosed luminal space, allowing unlimited access to the apical and basolateral sides of the barrier but preserving all the advantages associated with the use of this multicellular system [37]. Small et al. used the enteroid monolayer model derived from the human embryonic stem cell line H1 (WA01) to simulate human intestine for studying the interaction of *Escherichia coli* with the intestinal epithelium [35]. Moreover, using murine and human enteroid-derived monolayers, Sharma et al. studied the effects of nicotine-containing e-cigarettes on the intestinal barrier. It was found that the exposure to nicotine-free cigarettes reduced the expression of tight junction markers and increased inflammation in response to infections [38].

The generation of enteroids directly from donor tissue allows researchers to gain detailed information about a specific pathology [37]. For example, Meir and coworkers generated enteroids from gut samples obtained from patients with Crohn’s disease: this system was developed to provide an appropriate in vitro model to study the pathogenesis of barrier dysfunction in Crohn’s disease. The results demonstrated that patient-generated enteroids were able to maintain some characteristics of the intestinal intercellular junctions that are normally altered in Crohn’s disease [39], thus offering a promising in vitro model to test specific therapeutic agents. Similarly, Kourula et al. developed 3D enterocyte-enriched human duodenal and colonic organoids produced from biopsies of patients undergoing diagnostic colonoscopies. These enteroids were used to characterize the availability, metabolism, and safety profile of several small molecules. The results proved that the enteroids were able to distinguish between highly and poorly permeable compounds (e.g., ketoprofen, valacyclovir, propranolol, digoxin, atenolol), display metabolic activity for key drug-metabolizing enzymes (e.g., cytochrome P450 (CYP)3A4, CYP2C9, CYP2C19, CYP2D6, CYP2J2, uridine 5’-diphospho-glucuronosyltransferase (UGT)1A1, UGT2B7, and Sulfotransferase (SULT)1A), and accurately identify toxic drugs. It was also demonstrated that the toxicity of some drugs, such as gefitinib, can be revealed by intestinal barrier alterations, highlighting the value of organoids in predicting a drug’s safety profile [40]. Moreover, enteroids can be genetically manipulated to express specific traits in order to identify potential therapeutic agents [41]. For example, Haga et al. genetically manipulated stem cell-derived human intestinal enteroids to better understand the role of the fucosyltransferase 2 gene in the human norovirus infection: the generation by clustered regularly interspaced short palindromic repeats (CRISPR) technology of genetically identical enteroid lines that either express or lack fucosyltransferase 2 allowed them to demonstrate that this enzyme is crucial for human norovirus infection [42].

Although enteroids represent a clear advance over other conventional methods, this technology still has limitations. For example, these models fail to recreate the physiological environment of the intestinal tract, which includes supportive stromal cells and the microbial component [41]. Furthermore, enteroids accumulate materials secreted by goblet, enteroendocrine, and Paneth cells in their closed lumen: this is a non-physiological condition since these materials would be removed in vivo by peristaltic movements and the luminal flow [43]. Other limitations include the scarce scalability, reproducibility, and long-term culture of enteroids, as well as the difficulty to collect the appropriate source tissues [37,41].

Table 2 summarizes the 2D/3D cell-based models of the intestinal barrier described in this chapter.

## 4. Three-Dimensional Bioprinting

Three-dimensional bioprinting is a sub-category of additive manufacturing and an innovative technology in preclinical research. It offers the possibility to create de novo functional tissues and organs that closely replicate the physiological structure, complexity, and function of native tissues by using viable cells, biomaterials, and biological molecules (Figure 2) [44]. There are various 3D bioprinting techniques including inkjet-based printing, extrusion-based printing, and light-based printing, each with distinct advantages like automation, geometric freedom, high precision, and cellular complexity. Inkjet-based printing, also referred to as droplet-on-demand printing, utilizes thermal or acoustic forces applied to a nozzle to eject liquid droplets onto a controlled substrate. One limitation of the inkjet-based printing is that the bioink must first be in a liquid state and it later undergoes polymerization to generate a solidified structure: this method may only be applied to bioinks with low viscosity and low cell concentration. Extrusion-based printing overcomes this constraint as bioinks with high cell density and viscosity may be used thanks to a pressure-drive technology, which provides relatively low resolution while producing shear stress-induced cell damage. In light-based printing (which includes laser-assisted printing and stereolithography), photopolymerization is used to rapidly and selectively solidify photosensitive bioinks [45].

Despite their technological differences, in all bioprinting techniques bioinks are dropped layer by layer, with specific spatial arrangement. The choice of the bioink is crucial and depends on a variety of factors such as printability, mechanical properties, maturation time, degradability, and commercial availability as well as the capability to preserve cell viability and proliferation (for example, dense matrices and stiff materials can reduce cell survival).

To develop a more physiological 3D intestinal model, Macedo and colleagues used digital light projection stereolithography bioprinting and a bioink composed of gelatin methacryloil and poly (ethylene glycol) diacrylate, enriched with human intestinal fibroblasts [46]; intestinal epithelial cells (Caco-2 and HT29-MTX) were seeded on the surface of this bioprinted scaffold, forming a polarized monolayer with tight junctions, mucus secretion, and TEER values comparable to those of the human intestine. This 3D model, featuring villus-like structures, was compared with both a flat analog model and a conventional 2D co-culture, assessing functional and molecular parameters such as fibroblast viability and morphology, epithelial barrier formation, alkaline phosphatase activity, transporter expression, and compound permeability. The embedded fibroblasts showed high viability and the ability to secrete extracellular matrix. The 3D model exhibited higher TEER values, increased alkaline phosphatase activity, and reduced gene expression of efflux transporters such as P-gp and multidrug resistance-associated protein (MRP)2, alongside increased expression of MRP1. Permeability analysis using three model drugs (metoprolol, atenolol, and colchicine) demonstrated that the three-dimensional structure significantly influences absorption, particularly for compounds with low and moderate permeability. These findings confirmed the enhanced physiological relevance of the 3D villus-like model compared to traditional systems for studying intestinal absorption and transport [46].

Typically, engineered scaffolds are seeded with specific cell types, which may be fully differentiated (such as fibroblasts and endothelial cells) or have high proliferative and differentiative potential (like stem, pluripotent, or multipotent cells). Since cell lines such as Caco-2 and HT29 are derived from tumors, they tend to undergo genotypic changes during prolonged passages, potentially responding to stimuli in an altered manner compared to non-tumor cells [47]. To overcome this risk, Madden et al. developed a 3D bioprinted intestinal model using human primary intestinal epithelial cells and myofibroblasts to reproduce architecture and function of the native intestine [48]. Tumor necrosis factor (TNF)-α treatment activated an inflammatory response with morphologic alteration resulting in dissociation of cells from the interstitial layer, lactose dehydrogenase release, and upregulation of inflammatory genes (interleukin (IL)-6, IL-8, C-C motif chemokine ligand 2, C-X-C motif chemokine ligand 10, intercellular adhesion molecule 1), thus confirming the model’s utility for toxicity studies and inflammatory bowel diseases. Additionally, the model exhibited high expression of epithelial markers like E-cadherin and villin and markers for specialized cell types, such as Paneth cells (lysozyme), enteroendocrine cells (chromogranin A), and goblet cells (mucin-2). Moreover, key nuclear receptors (vitamin D receptor, pregnane X receptor, constitutive androstane receptor), major Phase I/II enzymes (CYP3A4, CYP2C9, glutathione S-transferase pi 1, UGT1A1), and essential intestinal transporters, including efflux (P-gp, breast cancer resistance protein (BCRP), uptake (PEPT1, Organic anion transporting polypeptide 2B1), and bile acid transporters (apical sodium dependent bile acid transporter, organic solute transporter alpha and beta), were expressed at levels comparable to native tissue, making this model ideal for evaluating metabolism and pharmacological interactions. In this 3D intestinal model, digoxin and topotecan were used to assess the functionality of the efflux transporters P-gp and BCRP, respectively, demonstrating active apical transport consistent with human intestinal tissue. Lucifer yellow and propranolol were used to evaluate paracellular and transcellular permeability, confirming the model’s ability to discriminate compounds based on their permeability profiles. Mitoxantrone, a low-permeability BCRP substrate, further supported the transporter functionality assessment. Moreover, in order to evaluate toxic responses, the authors used indomethacin, which induced a dose-dependent decrease in TEER and epithelial damage, highlighting the model’s sensitivity to barrier disruption [48].

Tofani et al. developed a 3D bioprinted intestinal model enriched with HT-29 (a human colon cancer cell line) and Caco-2 cells, and a collagen-based extracellular matrix. This model not only had a structure significantly more representative of the native intestinal mucosa compared to two manually fabricated models but also exhibited markedly higher levels of mucin-2 and key molecular markers related to intestinal function, such as claudin-2 and ATP-binding cassette sub-family B member 1. Furthermore, this bioprinted model displayed enhanced resistance to ibuprofen-induced damage, with better preservation of cellular viability and reduced disruption of the microvilli structure compared to the manually fabricated models [49].

Accurate in vitro reproduction of the human intestinal structure and function was achieved by Li et al., who successfully created a centimeter-scale intestine featuring a complex tertiary structure [50]. The authors developed a novel photocurable bioink made of methacrylated gelatin, methacrylated sodium alginate, and poly diacrylate, which was used to bioprint an intestine-like scaffold with large tube-like and ring folds. This hollow lumen scaffold was then seeded with a monolayer of Caco-2 cells, forming a polarized epithelium with crypt-villi domains to mimic the intestinal epithelial barrier. Additionally, endothelial cells from human umbilical vein proliferated and formed a microvascular network when encapsulated within the scaffold. This intestine-like construction may be suitable for various in vitro studies, such as drug testing, toxicity assessment, and microbiota interactions. To investigate the selective absorption capacity of the 3D bioprinted model, two fluorescein isothiocyanate (FITC)-dextrans of different molecular weights (4 kDa and 70 kDa) were employed. The results demonstrated that the low molecular weight dextran was able to permeate through the epithelial layer, whereas the high molecular weight dextran exhibited significantly reduced permeability. These findings confirm that the 3D model is capable of recapitulating the molecular selectivity characteristic of the native intestinal epithelium [50].

Although advancement in 3D bioprinting has led to the creation of more structurally complex and realistic intestinal models, significant challenges still remain to be addressed. One of them is the intrinsic cellular complexity of the organ, which contains a variety of highly specialized cells including enterocytes, goblet cells, Paneth cells, and fibroblasts, combined with the interactions between the immune system and the microbiome, making it particularly difficult to faithfully replicate the structure and function of the intestine in bioprinted models. Concurrently, a key aspect is the development of bioinks that must be not only stable and biocompatible but also capable of supporting cell growth and differentiation, while preserving the mechanical properties of the intestinal tissue, such as elasticity, resilience, and tensile strength [51]. In this regard, the design of scaffolds that mimic the intestinal microenvironment is critical to ensure proper support and organization of the cells. Moreover, the models should also take into account the dynamic behavior and plasticity of the intestine, whose homeostasis depends on mechanical forces such as peristaltic waves and blood flow and on villi motility [52]. Lastly, a significant goal to be achieved is the preservation of bioprinted models’ viability and function for extended periods, as many research applications require long-term experimentation, especially in pharmacological fields. Technological advanced models are using hydrogels or nanofibers to create villi-like scaffolds with micro-architectural features able to mimic the villi and crypt structure of intestinal surface. This provides a more realistic surface area, significantly improving the modeling of drug permeability [53].

Table 3 summarizes the 3D bioprinted models of intestinal barrier described in this chapter.

## 5. Organ-on-a-Chip

A remarkable step forward the in vitro representation of the human intestine is the so-called “chip-based in vitro organ model” (Figure 2) [54]. In the case of the intestine, chips have been developed with two different channels, separated by a porous extracellular matrix-coated membrane with both epithelial (on one side) and endothelial (on the other side) cell layers separately exposed to fluid flow [55]. The medium flows through micrometer-scale hollow channels lined with living human cells, and the cells cultured in a small-scale environment grow under conditions similar to in vivo. This allows researchers to use the organ-on-a-chip in studies aimed to assess the safety, efficacy, and absorption of drugs and active compounds [55,56,57]. Indeed, the advantage these systems have over traditional cell cultures mainly comes from the incorporation of microfluidics and the deriving physical forces like shear stress, cyclic strain, and mechanical compression [57]. Moreover, these microdevices are also suitable to set up hypoxia gradients that, combined with the dynamic flow, allow co-culturing human intestinal cells with a living complex microbiome, thus adding another fundamental element to appropriately mimic the in vivo condition [55].

In a recent work, Morelli and collaborators investigated the effects on permeability, morphology and inflammation caused by four different toxins (nigericin, patulin, ochratoxin A, and mellitin) on the intestinal epithelium by using a gut-on-a-chip model with Caco-2 tubules (OrganoReady^®^ Colon Caco-2) [58]. This system resulted to be promising for a proper multi-parametric analysis of molecule-specific alterations in barrier damage. However, as already recalled, Caco-2 cells carry mutations that could be inappropriate to represent normal intestinal functions; thus, working with biopsy-derived materials would be a valid option.

Consistently, Kasendra et al. combined two models, i.e., intestinal organoids and organ chips, to obtain an advanced system that ensured working with material derived from biopsies and therefore from healthy tissue [43]. In detail, they set up the organoid cultures exploiting intestinal crypts obtained from human intestinal endoscopic biopsies. Then, the organoids were enzymatically dissociated into cells subsequently seeded on the extracellular matrix-coated porous membrane to obtain the epithelial layer. The authors were able to obtain elongated villi-like structures lined with epithelial cells and characterized by a strong barrier function, secretion of mucus, apical enzymatic activity, and constant access to the fluids flow through the apical lumen. Moreover, the system ensured luminal flow both on the epithelial and vascular channels, as well as peristalsis-like cyclic deformations giving essential mechanical cues. The importance of the shear stress was pointed out also by Yin et al., who developed an organ chip to investigate cellular differentiation in response to physical mechanisms [59]. Fluid flow enhanced intestinal maturation/differentiation as demonstrated by the comparison of the proteomic profiles of enteroids placed in the intestine chip and their counterpart cultured as monolayers in the static environment of transwell inserts. Remarkably, a greater level of expression of villus protein, cell height, and number of microvilli were found, demonstrating the superiority of the dynamic environment over the static one.

This aspect has also been confirmed in a recent study by Gleeson and collaborators, where two different in vitro models, i.e., Caco-2 cells, cultured in a transwell or in a continuous flow microfluidic gut-on-a-chip (Emulate Bio, Inc., Boston, MA, USA) were used to test the effect of two intestinal permeation enhancers (sodium caprate and sucrose monolurate) potentially suitable to overcome low oral bioavailability [60]. Differences were found in the concentration of permeation enhancers necessary in the two models to induce an increase in permeability of dextran, insulin and octreotide, aligning the chip model more closely to the ex vivo and in vivo ones.

Moreover, gut-on-a-chips proved to be suitable to mimic human inflammatory conditions, such as the inflammatory bowel disease [61]. Regarding this, Marr and coworkers developed PREDICT96, a high-throughput microfluidic platform designed to accommodate 96 microscale devices on a single plate where human primary colon epithelial cells were cultured. Due to their role in inflammatory bowel disease etiology, interferon (IFN)-γ and TNF-α were used as barrier damage-inducing molecules, and their effects were evaluated in combinatorial dose curves [62].

The superiority of the gut-on-a-chip models over the static models described in the previous chapters is evident: the presence of biologically relevant mechanical cues thanks to the dynamic environment, the possibility to culture biopsy-derived materials, the ability to integrate the system with various sensors are just some of the advantages of these systems. However, even the gut-on-a-chip models cannot be considered without limitations [55]. First of all, these systems are characterized by a higher cost compared to the static 2D/3D cell culture models and require more complicated operations to set up the experiment [63]. In addition, these models still lack some components normally building up the intestinal barrier (e.g., the immune cells) [57,64]. Indeed, although these chips are able to provide mechanical cues, support microbiota growth, and establish oxygen gradients, models combining these three elements with intestine and immune cells to recreate an immuno-competent intestinal model are not available so far [65]. Finally, the already cited study by Morelli et al. underlined a crucial limitation of these kinds of in vitro models, i.e., an increased sensitivity to toxins [58]. Despite these limitations, researchers are developing emerging technologies to enhance the potential of this model. For instance, they developed next generation intestine-on-chip platforms incorporating crypt-villus structures, peristaltic motion, integrated biosensors for real time permeability and metabolic readouts, and fluid flow to mimic the peristaltic motion and shear stress of the gut lumen and capillary flow on the basal side [66]. In addition, alternative chips made of different materials, such as perfluoropolyether or cyclin olefin copolymer chips, have been developed to overcome the limitations of using polydimethylsiloxane, commonly employed in the fabrication of microfluidic devices.

Table 4 summarizes the organ-on-a-chip models of intestinal barrier described in this chapter.

## 6. Ex Vivo

Ex vivo intestinal models are biological systems consisting of tissue fragments removed from the intestine tracts and kept vital in a controlled laboratory environment (Figure 2). These models have the advantages of preserving the full cellular and extracellular composition, the natural 3D tissue structure, and the typical local microbiota of the native organism [57]. Ex vivo models therefore represent an intermediate step between in vitro (cultured cell-based models) and in vivo (whole-organism) studies.

Experimental work using human explants is obviously limited, but many ex vivo models set up using animal explants are suitable for human samples too. For this reason, in this chapter results obtained on non-human explants are also reviewed.

One of the most used systems for studying intestinal drug transport was originally set up by Wilson and Wiseman in 1954 [67]. In detail, a piece of small intestine from a rat or golden hamster was everted to have the intestinal mucosa in direct contact with the well-oxygenated suspending medium. After eversion, the intestinal piece was tied at both the ends, and the sac was filled with sufficient fluid to distend the wall and concentrate the transported substances to be measured. This model has the advantages of preserving the entire tissue structure and providing a large surface area to study the absorption process [67,68,69]. Nowadays, this ex vivo technique defined as “everted sac technique” is still widely exploited. As a recent example, a chicken ileum model of everted intestine was used to test the antidiabetic activity of silver nanoparticles from *Galinsoga parviflora* [70]. The results showed that silver nanoparticles inhibited key digestive enzymes and reduced glucose absorption, suggesting their use in antidiabetic therapies [70]. Similarly, Pol and collaborators used goat everted small intestine to examine the impact of orange (*Citrus aurantium dulcis*) peel extract on the intestinal absorption of aspirin, with positive results [71].

The Ussing chamber can also be used as an ex vivo model to assess the transport of ions, nutrients, and drugs across intestinal tissue [30,33,72] by measuring electrical potential difference [73,74]. Duarte et al. used Ussing chambers to examine the impact of the mycotoxin deoxynivalenol on jejunal explants of broiler chickens. Researchers exposed the chickens’ intestinal samples to either deoxynivalenol alone or deoxynivalenol with an anti-mycotoxin additive. Samples treated with deoxynivalenol were found to show signs of damage such as vacuolization, reduction in height of enterocytes, and lymphatic vessel dilation, while the anti-mycotoxin additive mitigated these detrimental effects [75]. Streekstra and coworkers used Ussing chambers as a model for investigating the permeability of different drugs (e.g., talinolol, rosuvastatin, enalaprilat, propranolol) across human adult and pediatric small intestinal tissues. A notable finding was that the efflux transport of talinolol by MRP1 and rosuvastatin by BCRP was significantly higher in adult tissues compared to pediatric tissues, suggesting age-related differences in the expression or function of these key drug transporters [76].

One of the main advantages of using ex vivo models is that the tissue preserves a reactivity quite similar to that observed in vivo, even though it is no longer inside the organism. In fact, several works proved that ex vivo models of different tissues, such as prostate, skin, intestine, were able to preserve tissue architecture as well as physiological barrier function, signaling pathways, biological mechanisms, and cellular responses [77,78,79]. The use of ex vivo models for preclinical research also represents an ethical choice, since it allows researchers to replace animals with biopsies from human subjects or, in the case of animal samples, to reduce the number of animals needed for the experimentation, thus meeting two of the three requirements of the 3R principle [4]. However, the time window during which the explants maintain the original structural and functional features is limited, and a great goal would be to find a way to preserve the explants for a long time, delaying the deterioration of the tissue. A possible solution could be to replace a static system with a fluid dynamic one, which would ensure optimal oxygen and nutrient levels while also removing catabolites. In this regard, Amirabadi and collaborators developed a model of intestinal barrier based on a microfluidic chip called “intestinal explant barrier chip” that may host human and porcine colon tissue explants subjected to two separate fluid flows on both the luminal and basal side [27]. This system effectively maintained the viability, integrity, and permeability of tissue explants for up to 24 h: the different (neural, epithelial, and immune) cell populations were maintained; the crypts, submucosa, and muscular layers retained their structure, and the two fluid flows remained separate. Then, the model was used to assess the role of collagen damage in the intestinal barrier disruption using mouse intestine explants. Briefly, bacterial collagenase was added to the fluid flowing in the luminal channel, thus inducing dose-dependent alterations in collagen-1 and claudin-1, as well as evident damage of the epithelial layer [57].

Working with tissue explants implies significant challenges including limited sample availability, difficulty in finding suitable devices for long-term viability, and the risk of damage during handling. However, ex vivo models reproduce more accurately than all other models the in vivo conditions, making them a valuable tool to bridge the gap between in vitro and in vivo research [80].

Table 5 summarizes the ex vivo models of the intestinal barrier described in this chapter.

## 7. Future Perspectives

Although the in vitro models described in the present review proved to be suitable for the advancement of preclinical research related to the intestinal barrier, it is evident that there is still room for further development and improvement to establish a cutting-edge and highly predictive system. Addressing the limited translatability of pharmacokinetic, efficacy, and toxicity data from preclinical models to clinical settings is crucial to reverse the prevailing trend whereby more than 90% of investigational drugs fail during clinical development [81]. A clear improvement for the representation of the complex intestinal barrier in laboratories would be the consolidated integration of the intestinal microbiota in the in vitro systems. In this sense, the model developed by Jalili-Firoozinezhad and coworkers should be cited to point out its potential [82]. Indeed, they integrated Caco-2 intestine chips with microscale oxygen sensors and put the device in an anaerobic chamber to define a physiologically pertinent oxygen gradient across the human intestinal epithelium and microvascular endothelium made of human intestinal microvascular endothelial cells, which were cultured in parallel and isolated from each other by a membrane embedded with a porous matrix. They found that the presence of this transluminal hypoxia gradient enhanced the intestinal barrier integrity and maintained a biologically meaningful level of microbial diversity with both aerobic and anaerobic communities. After that, they co-cultured primary human ileal epithelium with a gut microbiota in this “hypoxic intestine chip”, noticing an enhanced epithelial barrier function compared to aerobic chips. This microfluidic system is very interesting since it may be used to model different regions of the intestine by culturing region-specific cells and by setting appropriate oxygen tensions suitable for a region-specific microbiome.

Beside the integration of the microbiota, the advantages associated with dynamic systems over static ones, such as the introduction of shear stress, should encourage researchers to develop such models. Among these platforms, millifluidic systems have begun to receive particular attention due to their reduced complexity and production costs if compared to chip-based models [14]. For instance, Colombo and coworkers tested the different intestinal absorption of caffeic acid, quinic acid, rosmarin acid, quercetin, and rutin exploiting Caco-2 cells cultured in a commercial multi-compartmental modular chamber together with the millifluidic LiveFlow^®^ bioreactor, demonstrating its predictive potential towards the in vivo environment [14]. Another recent model was presented by Almalla et al., who developed a 3D hydrogel scaffolds-integrated printed millifluidic tissue chamber where human intestinal stem cells were cultured. This resulted in multi-lineage differentiation (enterocyte and goblet cell lineages) thanks to the combination of the 3D environment provided by the biomimicking scaffold and the physiological shear stress [83].

A shared strength of these models lies in their modular nature, which makes them highly versatile and suitable for generating a variety of experimental conditions. However, validating complex dynamic models remains a major open issue in the field. Meanwhile, given its demonstrated potential, artificial intelligence is increasingly regarded as a transformative force in research, with the capacity to significantly impact future developments. The convergence of in vitro models and artificial intelligence holds promise for accelerating research breakthroughs by facilitating the creation of predictive models, improving the fidelity of disease modeling, and supporting the advancement of personalized therapeutic approaches [84].

## 8. Conclusions

The need for highly predictive models of human intestine to accelerate the preclinical research and limit the use of laboratory animals has pushed—and it is still pushing—scientists and engineers to develop various in vitro systems. Although the ideal model should properly reproduce the intestine structure and function in either health or disease, in the experimental practice the models so far set up have aimed at mimicking specific intestinal features related to the molecules or processes under investigation. Artificial membranes are useful to study passive absorption; the more complex systems involving 2D/3D cell cultures are suitable to reproduce the transcellular pathway; the highly technological organs-on-a-chip, by mimicking the in vivo cellular and mechanical complexity, allow researchers to identify the multiple factors involved in molecular interactions with the intestinal barrier; intestine explants replicate in full the native organ under controlled conditions, thus providing the most comprehensive in vitro model. All these models have advantages and disadvantages, as discussed in the previous chapters and summarized in Table 6, but all have given important contribution to advance the knowledge on the interaction of drugs, toxins, and xenobiotics with the intestinal barrier. In a future perspective, the inventiveness of scientists and progress in technology will allow overcoming the current limitations and setting up refined in vitro models of the human intestine for faster, more standardized, and effective studies on gut pathophysiology and therapeutic treatments.

## Figures and Tables

**Table 1:** Artificial membrane-based models described in Section 2.

Experimental Model	Investigated Molecules	Applications	Results	References
PAMPA	parabens, bisphenols, isothiazolinones, phthalates	To test the permeability of chemical compounds	Log P is a key factor in permeability, but also molecular weight, pKa, chemical structure, and interactions with membrane lipids also influence permeation	[15]
PAMPA	DNA gyrase and topoisomerase IV inhibitors	To estimate the passive gastrointestinal absorption of 13 DNA gyrase inhibitor compounds	PAMPA showed low permeability for acidic compounds and high permeability for neutral and more lipophilic compounds	[19]
real-time PAMPA	1,2-dioleoyl-sn-glycero-3-phosphocholine (DOPC), DOPC + stearic acid	To propose a new RT-PAMPA method for real-time measurement of passive drug permeability using fluorescent artificial receptors	The method quickly distinguishes drugs with different permeabilities, is compatible with various solvents and membranes, and improves efficiency compared to traditional PAMPA	[18]
PAMPA and Caco-2 cells	polar *N*-methylated cyclic hexaalanines, lipophilic tri-*N*-methylated Veber–Hirschmann peptides, lipophilic peptide	To study pairs of enantiomeric peptides of 3 polar peptides and 2 lipophilic peptides to investigate the possible involvement of stereospecific biological transporters	The spatial structure of peptides has a significant influence on their intestinal permeability through carrier-mediated transporters	[20]
AMI-system and Caco-2 cells	Carvedilol, felodipine, fenofibrate, ibuprofen, indomethacin, itraconazole, JNJ39393406, ketoconazole, metroprolol, naproxen, piroxicam, posoconazole, tadalafil, zafirlukast	To evaluate AMI as a rapid, cost-effective, and simple alternative to the Caco-2 cell model for estimating the passive intestinal permeability of poorly soluble drugs	The AMI system demonstrated a high correlation with the Caco-2 model in assessing passive intestinal permeability, representing a rapid, cost-effective, and simple alternative, although it does not replicate active transport or the mucus barrier	[21]
DAMPA	Atenolol, carbamazepine, cimetidine, ketoprofen, metoprolol, piroxicam, propranolol, acyclovir, alprenolol, antipyrine, chlorotiazide, famotidine, methotrexate, nadolol, pindolol, salicyclic acid, sulfas atrazine, sulpiride, terbut aline, warfarin	To develop and validate the DAMPA method for predicting human intestinal drug permeability in vitro	DAMPA showed a strong correlation with human data representing an efficient and accurate alternative to traditional cell-based models	[16]
PAMPA + probiotic bacteria and bile acids	gliclazide	To estimate the influence of bile acids and intestinal bacteria on intestinal absorption of gliclazide	pH, probiotics, and bile acids affect the permeability and bioavailability of gliclazide	[22]
PAMPA + probiotic bacteria and deoxycholate	azathioprine	To evaluate the permeability of azathioprine at different pH levels, both alone and in combination with deoxycholic acid and probiotics	Azathioprine shows higher permeability at acidic pH; probiotics increase its permeability but reduce its overall amount, while deoxycholic acid decreases permeability	[23]

**Table 2:** Two-dimensional and three-dimensional cell-based intestine models described in Section 3.

Experimental Model	Investigated Molecules	Applications	Cell Source	Culture Duration	Results	References
Transwell membrane (cell monolayer)	Epigallocatechin gallate (EGCG)	To develop phosphatidylcholine-based nanoparticles to enhance the oral bioavailability of EGCG and investigate its intestinal absorption using Caco-2 cell models	Human	Up to 72 h	The nanoparticles improved digestive stability, promoted cellular uptake, and inhibited efflux, thereby enhancing overall EGCG absorption	[29]
Ussing chamber	Hesperidin	To evaluate whether pretreatment with hesperidin mitigates X-ray (2 Gy)-induced damage to intestinal barrier function	Human	Up to 72 h	Hesperidin pre-treatment protected intestinal barrier integrity from X-ray damage by improving cell survival, reducing permeability, and restoring tight junction proteins	[31]
Ussing chamber	Polyphenols	To study the transport, metabolism, and effect of apple polyphenols on the intestinal barrier, particularly on tight junctions and the transepithelial resistance of cells	Human	Up to 24 h	Apple polyphenols improve the intestinal barrier by increasing transepithelial resistance and stimulating tight junction proteins, also promoting epithelial damage repair	[32]
Transwell membrane (co-culture)	Silicon quantum dots and iron oxide nanoparticles	To develop an in vitro intestinal model to study nanoparticle transport	Human	Up to 21 days	The nanoparticles did not cross the model due to aggregation	[34]
Enteroid cell model	*Escherichia coli*	To develop an in vitro human intestinal model to analyze infection by *E. coli* O157:H7 and compare it with probiotic and commensal strains	Human	Up to 15.5 days	The model remained intact with apical saline. Non-pathogenic *E. coli* strains did not damage the epithelium, while *E. coli* O157:H7 caused loss of barrier integrity, strong bacterial adhesion, epithelial damage, and bacterial translocation after 24–36 h	[35]
Enteroid cell model	E-cigarette	To analyze the effects of chronic exposure to e-cigarette aerosols (with or without nicotine) on the intestinal barrier, using murine models and human organoids	Murine and human	Up to 24 h	Prolonged use of e-cigarettes damages the intestinal barrier, increases inflammation, and heightens susceptibility to bacterial infections	[38]
Enteroids from donor tissue	Ketoprofen, valacyclovir, propranolol, digoxin, atenolol	To evaluate the use of human intestinal organoids as in vitro models for studying drug absorption, metabolism, and intestinal toxicity	Murine and human	Up to 21 days	Intestinal organoids represent an effective and translational model for absorption, distribution, metabolism, and excretion, and intestinal safety studies	[40]
Enteroid cell model	Fucosyltransferase 2 (FUT2) gene	Study the role of the FUT2 gene in susceptibility to human norovirus infection using genetically modified human intestinal organoids	Human	Up to 5 days	FUT2 is necessary and sufficient to enable human norovirus to infect intestinal cells, affecting viral binding and replication	[42]

**Table 3:** Three-dimensional bioprinted intestine models described in Section 4.

Experimental Model	Investigated Molecules	Applications	Results	References
3D biopinted intestine model enriched with human intestinal fibroblasts. Intestinal epithelial cells (Caco-2 and HT29-MTX)	metoprolol, atenolol, colchicine	To create a 3D in vitro model of human small intestine that includes villus architecture and stromal compartment to better study drug absorption	The 3D model exhibits barrier and permeability functions more similar to the human intestine compared to flat models	[46]
3D bioprinted intestine model with human primary intestinal epithelial cells and myofibroblasts	digoxin, topotecan, lucifer yellow, propranolol, mitoxantrone, indomethacin	To develop a 3D human small intestine model using primary cells to improve preclinical prediction of drug absorption, metabolism, transport, and toxicity	The model replicates intestinal architecture and function with physiological barrier properties, active expression of metabolic enzymes and transporters, and realistic responses to drugs and toxins	[48]
3D bioprinted intestine model enriched with HT-29 and Caco-2 cells	ibuprofen	To create a 3D bioprinted intestinal model for preclinical drug testing studies	The bioprinted model containing fibroblasts replicates more accurately the human intestine compared to a 3D model without fibroblasts and traditional 2D models	[49]
3D bioprinted intestine-like scaffold with large tube-like and ring folds enriched with Caco-2 cells and human umbilical vein endothelial cells	FITC-dextrans (4 kDa and 70 kDa)	To develop a realistic and large-scale 3D in vitro intestinal model that faithfully reproduces the complex anatomical structure of the human intestine, including hollow lumen, folds, crypts, villi, microvilli, and capillary network	A vascularized intestinal tissue populated with epithelial and endothelial cells, and capable of mimicking the intestinal barrier was created	[50]

**Table 4:** Organ-on-a-chip intestine models described in Section 5.

Experimental Model	Investigated Molecules	Applications	Cell Source	Culture Duration	Results	References
OrganoReady^®^ Colon Caco-2	Nigericin, patulin, ochratoxin A, mellitin	To evaluate the effects of different enterotoxins on the intestinal epithelial barrier	Human	Up to 8 days before toxin exposure	The model showed greater sensitivity compared to conventional models, allowing the differentiation of the toxins’ pathogenic mechanisms in terms of permeability, cytotoxicity, and cell morphology	[58]
Primary human intestine chip	Lucifer yellow	To provide a more realistic and functional tool for studying intestinal physiology, cellular interactions, intestinal diseases, pharmacology, and personalized medicine	Human	Up to 12 days	The chip recapitulates key structures such as intestinal villi, maintains a functional epithelial barrier, and reproduces multilineage cell differentiation; it provides a more sensitive and relevant system compared to 2D models	[43]
Emulate Bio, Inc.	Sodium caprate and monolaurate with dextran, insulin and octreotide	To evaluate the effectiveness of intestinal permeation enhancers to improve the oral delivery of peptide drugs	Human	Up to 21 days	The model shows more realistic permeability and greater resistance to bile salts compared to a transwell model, making it a useful tool for the development of oral peptide drugs	[60]
PREDICT96	IFN-γ, TNF-α	To study the colonic epithelial barrier, assessing damage and recovery in response to inflammatory cytokines relevant to inflammatory bowel disease	Human	Up to 14 days	Exposure to the inflammatory cytokines TNF-α and IFN-γ induced dose-dependent barrier damage, with variations in sensitivity among donors	[62]

**Table 5:** Ex vivo intestine models described in Section 6.

Experimental Model	Investigated Molecules	Applications	Results	References
Chicken everted intestine	Silver nanoparticles from *Galinsoga parviflora*	To evaluate the in vitro antidiabetic activity of silver nanoparticles from *G. parviflora* by analyzing their inhibition of α-amylase and α-glucosidase, as well as glucose uptake using the everted gut sac method with chicken ileum	Silver nanoparticles of *G. parviflora* showed strong in vitro antidiabetic activity, outperforming Acarbose in inhibiting α-amylase and α-glucosidase enzymes, and reducing short-term glucose absorption, indicating potential for managing postprandial blood glucose	[70]
Goat everted intestine	*Citrus aurantium dulcis* peel extract with aspirin	To evaluate how orange peel extract can improve the intestinal absorption of aspirin	The flavonoids present in orange peel extract enhanced its ability to improve aspirin absorption	[71]
Chicken intestine explants in Ussing chambers	Deoxynivalenol (DON)	To study the toxic effects of the mycotoxin DON on the intestinal barrier and to evaluate the efficacy of an antimycotoxin additive in protecting it	The mycotoxin DON rapidly damaged the integrity of the intestinal epithelium, and the tested antimycotoxin additive mitigated these damages by reducing cytoplasmic vacuolization and apoptosis	[75]
Human intestine explants in Ussing chambers	Talinolol, rosuvastatin, enalaprilat, propranolol	To study intestinal drug absorption in children and compare it to adults	Passive permeability is similar to that of adults, while active transport may be reduced in younger children	[76]
Mouse intestine explants in microfluidic chip	Collagen-1, claudin-1	To evaluate the alterations induced by bacterial collagenase on the integrity and permeability of the intestinal barrier	Collagenase increases intestinal permeability, damages tight junction proteins, alters collagen structure, and modifies goblet cells by reducing mucus production, thereby compromising the barrier	[57]

**Table 6:** Advantages, limitations, and primary applications of intestine in vitro models.

In vitro Models	Advantages	Limitations	Primary Applications
Artificial membranes	Inexpensive, easy to use, and effective for high-throughput screening	Lack living cells, do not reproduce tissue complexity, and paracellular and active transports	Study of passive transport and molecular diffusion
2D cultures	Easy to culture and manipulate, relative low cost, and good reproducibility	Lack 3D structure and interactions between the different intestinal cell types	Toxicity testing and absorption studies
3D cultures	Better representation of tissue structure and interactions between different cell types than 2D cultures	More costly, generally with an enclosed luminal space, limited scalability, reproducibility, and long-term culture	Toxicity testing and absorption studies in a barrier with a more realistic functionality
3D Bioprinting	Precise cell–matrix arrangement and possibility to develop personalized models	Expensive, complex technology that requires specialized operators	Reconstruction of complex tissues, tissue regeneration, drug testing, toxicity, and microbiota interactions
Organ-on-a-chip	Microfluidic environment with all the derived physical stimuli, real-time monitoring	Technical complexity, high costs and lack of immune cells	Assessment of safety, efficacy, and absorption of drugs and active compounds
Ex vivo	Original architecture very close to biological reality	Limited lifespan and high inter-donor variability	Pharmacological and physiological studies on human or animal tissues

## Data Availability

No new data were created or analyzed in this study. Data sharing is not applicable to this article.
